# Parkinson’s Disease: Immunometabolic Control Points Across Neural, Vascular, and Peripheral Systems

**DOI:** 10.1155/padi/5349481

**Published:** 2026-08-17

**Authors:** Felix Y. Narvaez Irizarry, Alondra M. Bermudez Adorno, Boris Ermolinsky, Lilia Kucheryavykh, Mikhail Inyushin

**Affiliations:** ^1^ Universidad Central del Caribe (UCC), Bayamón 00960, Puerto Rico, USA; ^2^ University of Texas Rio Grande Valley (UTRGV), Brownsville 78539, Texas, USA

## Abstract

Parkinson’s disease (PD) is a progressive neurodegenerative disorder most associated with degeneration of dopaminergic neurons in the substantia nigra pars compacta. Increasing clinical and experimental evidence, however, indicates that PD is a multisystem disease in which immune, metabolic, vascular, and peripheral nervous system dysfunction precede and shape motor circuit failure. Nonmotor manifestations, including olfactory impairment, sleep and autonomic disturbances, gastrointestinal dysfunction, and cognitive decline, often arise years before motor diagnosis, highlighting pathogenic mechanisms beyond dopamine deficiency alone. In this review, we synthesize evidence supporting the view that PD represents a disorder of chronic immunometabolic dysregulation rather than isolated neuronal loss. We focus on underestimated but high‐impact immunological control points that integrate central and peripheral disease mechanisms, including environmental and microbial immune priming, gut–brain axis interactions, platelet‐mediated inflammatory signaling, amyloid‐β (Aβ) as an innate immune peptide, immune checkpoint regulation via the PD‐1/PD‐L1 axis, and nicotinamide adenine dinucleotide (NAD^+^) homeostasis governed by nicotinamide phosphoribosyltransferase (NAMPT). Recent pharmacological evidence further supports this framework, as metabolic interventions that improve systemic energy balance and reduce inflammation have also demonstrated neuroprotective effects in experimental models, reinforcing the concept that immunometabolic pathways are therapeutically actionable. Motor impairment in PD arises from basal ganglia network dysfunction, accompanied by pathological beta‐band synchronization, which is increasingly linked to neuroinflammation and metabolic stress. In parallel, platelet activation during inflammation and vascular injury releases amyloid precursor protein and Aβ, linking systemic immune activation to neurovascular signaling. Clinical observations from immune checkpoint inhibitor therapy demonstrate that disruption of PD‐1/PD‐L1 signaling can lead to Parkinsonism and neuroinflammatory syndromes, underscoring the importance of immune restraint in neural homeostasis. Finally, we highlight NAMPT‐dependent NAD^+^ salvage as an important immunometabolic hub integrating energy metabolism, inflammation, and neuronal survival. Together, these findings support a unifying framework in which PD reflects failure of immunometabolic control across neural and peripheral systems, suggesting new avenues for disease‐modifying therapeutic strategies beyond symptomatic dopamine replacement.

## 1. Introduction

Parkinson’s disease (PD) is a progressive neurodegenerative disorder traditionally defined by the loss of dopaminergic (DA) neurons in the substantia nigra pars compacta (SNc). However, growing clinical, pathological, and experimental evidence shows that PD affects multiple body systems, including the immune, metabolic, vascular, and peripheral nervous systems, long before typical motor symptoms appear. Although diagnosis remains anchored to motor features such as bradykinesia, rigidity, tremor, and postural instability, nonmotor manifestations, including olfactory dysfunction, sleep disturbances, autonomic failure, neuropsychiatric symptoms, and cognitive decline, often precede motor onset by years and substantially contribute to disease burden and reduced quality of life [1–4].

From an immunological perspective, PD can be understood as a disorder of chronic, dysregulated neuroimmune activation rather than isolated neuronal loss. Innate and adaptive immune pathways are engaged early, with persistent microglial activation, peripheral immune involvement, altered cytokine signaling, vascular dysfunction, and metabolic stress shaping disease progression [5, 6]. These processes interact with classical basal ganglia circuitry dysfunction, amplifying network instability and rendering DA neurons particularly vulnerable to energetic failure, inflammatory injury, and programmed cell death [7–9]. Recent pharmacological studies further support the concept that systemic metabolic interventions can influence neurodegenerative processes. Sodium–glucose cotransporter‐2 (SGLT2) inhibitors, such as empagliflozin, exert pleiotropic effects beyond glycemic control, including attenuation of systemic inflammation, reduction of oxidative stress, and improvement of mitochondrial function [10]. Experimental work further indicates that SGLT2 inhibition can improve cerebral mitochondrial efficiency and reduce neuroinflammation in models of brain injury and metabolic stress [11, 12]. These observations support the broader concept that metabolic reprogramming can stabilize neuroimmune homeostasis and may represent a viable disease‐modifying strategy in PD and related neurodegenerative conditions.

The aim of this review is to identify and integrate novel and insufficiently investigated control points in PD pathogenesis, with particular emphasis on immunological and immunometabolic mechanisms that link central and peripheral systems. Instead of repeating well‐known DA replacement strategies, we focus on regulatory mechanisms that influence disease progression across multiple scales, including immune checkpoints, platelet‐driven inflammation, amyloid‐β (Aβ) as an immune‐responsive molecule, microbiome effects on immunity, and nicotinamide adenine dinucleotide^+^ (NAD^+^)‐dependent metabolism controlled by nicotinamide phosphoribosyltransferase (NAMPT). By highlighting these underexplored control points, this review seeks to provide a conceptual framework for disease‐modifying strategies that extend beyond symptomatic motor relief and address the upstream immune and metabolic drivers of neurodegeneration.

## 2. Neuroimmune Modulation of Basal Ganglia Networks and Sensory Signatures in PD

Motor impairment in PD arises from dysfunction of basal ganglia–thalamocortical circuits that normally integrate excitatory glutamatergic and inhibitory GABAergic signaling under DA modulation [13]. In the healthy state, dopamine acting on D1 receptors facilitates the direct pathway (striatum ⟶ globus pallidus internus (GPi)/substantia nigra pars reticulata(SNr) ⟶ thalamus), while dopamine acting on D2 receptors suppresses the indirect pathway (striatum ⟶ external globus pallidus (GPe) ⟶ subthalamic nucleus (STN) ⟶ GPi), maintaining balanced thalamocortical drive. In PD, loss of SNc DA input shifts this balance toward excessive indirect pathway activity, increased STN excitation, and pathologically elevated inhibitory output from the GPi and SNr [13, 14]. Importantly, this circuit dysfunction does not occur in isolation but is accompanied by abnormal synchronization of neuronal populations, particularly exaggerated beta‐band oscillations (13–35 Hz), which correlate with rigidity and bradykinesia and reflect maladaptive network‐level immune–metabolic stress responses [1, 15]. Clinically, PD progression is commonly described using the Hoehn–Yahr staging system, which captures the transition from unilateral motor signs to severe postural instability and immobility [16].

Neuroinflammation is increasingly recognized as a central amplifier of this circuit pathology [7, 8]. Activated microglia respond to α‐synuclein aggregates, mitochondrial damage, and neuronal stress signals, resulting from neuronal damage in PD, by releasing cytokines such as interleukin‐1β (IL‐1β), TNF‐α, and interleukin‐6 (IL‐6), which further impair synaptic function and promote oscillatory instability. These inflammatory signals interact with glial metabolic pathways and vascular elements, linking immune activation to energy failure and impaired neurotransmission. Together, these observations indicate that inflammatory signaling and metabolic dysfunction are tightly coupled in PD, consistent with an immunometabolic mode of neurodegeneration [5, 17]. In addition to classical DA and glutamatergic mechanisms, purinergic and adenosinergic signaling pathways play critical roles in regulating both neurotransmission and neuroinflammation within basal ganglia circuits. Adenosine A2A receptors, highly expressed in striatal neurons, modulate DA signaling and influence motor output as well as inflammatory responses, forming the basis for A2A receptor antagonists as therapeutic agents in PD [18–20]. In parallel, P2X7 receptors, expressed predominantly on microglia, function as sensors of extracellular ATP released during cellular stress and injury. Activation of P2X7 receptors promotes inflammasome assembly, IL‐1β release, and amplification of neuroinflammatory responses, thereby linking metabolic stress signals to circuit dysfunction [21, 22]. Together, these purinergic pathways represent important interfaces between neuronal activity, immune signaling, and metabolic state.

Clinically, this manifests not only as bradykinesia and rigidity but also as progressive gait impairment, freezing of gait, and reduced arm swing—often asymmetric and detectable early—reflecting failure of automatic motor programs under inflammatory stress [23, 24].

Current motor therapies act on specific “control points” within this immunometabolic network. Levodopa replenishes dopamine but does not address inflammatory or immune drivers [25]. Adjunctive therapies, such as dopamine agonists, catechol‐O‐methyltransferase (COMT) inhibitors, and monoamine oxidase‐B (MAO‐B) inhibitors, modulate dopamine availability, with MAO‐B inhibition particularly relevant given MAO‐B expression in astrocytes and its role in dopamine and reactive oxygen species metabolism [26]. Amantadine reduces levodopa‐induced dyskinesia in part by attenuating N‐methyl‐D‐aspartate (NMDA) ionotropic glutamate receptor–mediated glutamatergic excitotoxicity and downstream inflammatory signaling [27]. Deep brain stimulation (DBS) of the STN or GPi further illustrates an immunological dimension: rather than simply silencing neurons, DBS suppresses pathological beta synchrony and reduces network entropy (a quantitative measure of how irregular, unpredictable, or desynchronized neuronal activity is within a circuit, particularly in the basal ganglia during Parkinsonian states), indirectly dampening inflammatory circuit instability [28, 29]. Adaptive DBS systems now leverage real‐time subthalamic beta‐band oscillations (13–35 Hz), beta burst dynamics, and phase–amplitude coupling as control signals, integrating network‐level biomarkers that are shaped by inflammatory and metabolic influences on basal ganglia circuitry [30, 31]. In addition to DBS, lesion‐based network interventions such as focused ultrasound thalamotomy targeting the ventral intermediate nucleus provide symptomatic relief in tremor‐dominant PD, further underscoring the role of circuit‐level modulation rather than cell replacement alone [32]. Recent network‐level work proposes that the cortical “control” system—somato‐cognitive action network (SCAN) is abnormally hyperconnected with subcortical regions in PD, and that clinical improvement across pharmacologic and neuromodulation therapies is associated with reduction of this hyperconnectivity—consistent with the idea that targeting a network control node can rebalance distributed influences rather than modulating a single motor area [33].

Beyond motor symptoms, PD is defined by early nonmotor immune signatures. Olfactory dysfunction reflects early α‐synuclein pathology in the olfactory bulb and anterior olfactory nucleus, regions with direct environmental exposure and immune surveillance, supporting Braak’s hypothesis of peripheral entry and prion‐like propagation [34–36]. Olfactory dysfunction is not unique to PD and is also a well‐recognized early feature of Alzheimer’s disease, often preceding cognitive symptoms by years [34, 37]. In Alzheimer’s disease, it is associated with early tau pathology and, variably, Aβ deposition within limbic olfactory networks [37, 38]. Sleep disturbances, particularly REM sleep behavior disorder, represent another early failure of brainstem inhibitory circuits and strongly predict future synucleinopathy, again implicating immune‐mediated vulnerability of these nuclei [39]. Other early symptoms, such as autonomic dysfunction, gastrointestinal dysmotility, and constipation, further highlight early involvement of the enteric nervous system and gut‐associated immune tissue, linking PD to chronic peripheral inflammation [40, 41]. Bulbar involvement leads to hypophonia, dysarthria, and dysphagia, contributing to malnutrition and aspiration risk in advanced disease stages [42].

Accumulating evidence indicates that PD is associated with a disease‐specific body odor. Chemical analyses of skin sebum, the lipid‐rich secretion of sebaceous glands, have identified distinct volatile organic compound (VOC) profiles in PD patients that differ reproducibly from those of healthy individuals [43, 44]. These VOC signatures are sufficiently robust to be detected by trained dogs, which can distinguish PD patients from healthy individuals based solely on odor samples [45]. Sebum production and composition are strongly influenced by immune activity, oxidative stress, and lipid peroxidation, all of which are specifically altered in PD [46]. Sebaceous glands are immunologically active structures whose sebocytes express cytokine and pattern‐recognition receptors and participate in innate immune responses while shaping interactions with the skin microbiota [47, 48]. Alterations in immune or metabolic state can modify sebum composition, promoting microbial conversion of lipid substrates into volatile metabolites that contribute to disease‐specific odor profiles [49, 50]. It is present in many other metabolic disorders as well. Importantly, these sensory alterations likely reflect downstream consequences of systemic immunometabolic changes. Inflammatory signaling and oxidative stress can alter lipid metabolism in sebaceous glands, leading to shifts in sebum composition and downstream microbial processing into volatile metabolites [43, 46]. Similarly, inflammatory and metabolic disturbances within olfactory pathways may impair odor discrimination while also modifying local biochemical environments [34, 37]. These observations support the view that olfactory dysfunction and disease‐specific odor signatures are not merely ancillary features but integrated readouts of systemic immune and metabolic dysregulation.

From a network perspective, basal ganglia dysfunction, olfactory impairment, and disease‐associated body odor can be viewed as parallel readouts of a shared immunometabolic disturbance. Early immune activation in olfactory and peripheral tissues may prime central immune responses, lowering the resilience of basal ganglia circuits to metabolic stress and facilitating the propagation of α‐synuclein pathology. Conversely, progressive neuroinflammation within motor circuits feeds back to peripheral systems via autonomic and vascular pathways, reinforcing systemic immune dysregulation.

Together, these observations suggest that sensory abnormalities, particularly olfactory deficits and disease‐specific odor production, are not epiphenomena but integral components of PD pathophysiology. They reflect early and ongoing neuroimmune activity that modulates basal ganglia network stability and may serve as accessible biomarkers of underlying immunological control points. Targeting these control points offers an opportunity to intervene upstream of irreversible neuronal loss, potentially altering disease trajectories before classical motor symptoms become dominant.

## 3. Microbial, Viral, and Environmental Immune Priming as Potential Causal Drivers of PD

PD is not primarily a genetic disorder. Although mutations in genes such as SNCA, LRRK2, PINK1, and Parkin can cause familial forms of Parkinsonism, these account for only a small minority of cases. Pathogenic mutations account for approximately 5%–10% of PD, and even the most common genetic risk variants exhibit incomplete penetrance, indicating that additional nongenetic factors are required for disease expression [4, 51]. Twin studies further support this conclusion: concordance rates for PD are relatively low, and heritability estimates cluster around 30%, implying that the majority of disease risk arises from environmental and lifestyle influences rather than inherited genetics [52, 53]. These observations strongly suggest that PD develops through interactions between genetic vulnerability and external immune, metabolic, and environmental stressors.

Within this context, increasing attention has focused on the gut microbiome, infectious exposures, and environmental toxins as potential upstream contributors to PD pathogenesis. Rather than acting as single causative agents, microbes and viruses are thought to function as chronic immune primers that shape systemic and central nervous system inflammation over decades. The gastrointestinal tract represents a major immunological interface, containing extensive gut‐associated lymphoid tissue and maintaining constant contact with commensal and pathogenic microorganisms. Disruption of gut microbial homeostasis (dysbiosis) is increasingly recognized in PD and is associated with increased intestinal permeability, systemic inflammation, and altered microbial metabolites—including short‐chain fatty acids, lipopolysaccharide, hydrogen sulfide, bacterial amyloids, and bile acid derivatives—capable of affecting neuronal survival and mitochondrial function [54, 55]. Mechanistically, environmental toxins and peripheral inflammatory triggers converge on common pathways linking peripheral exposure to central neurodegeneration, including mitochondrial dysfunction, oxidative stress, and activation of innate immune signaling [56, 57]. These processes promote the release of damage‐associated molecular patterns that activate microglia and induce metabolic reprogramming toward a proinflammatory phenotype characterized by increased glycolysis and cytokine production [58, 59]. This metabolic–immune shift amplifies neuronal vulnerability, particularly in high‐energy‐demand DA neurons, thereby linking environmental exposure to progressive basal ganglia dysfunction.

Experimental evidence supports a causal role for peripheral immune dysfunction in driving Parkinson‐like pathology. In genetically susceptible Pink1^−^/^−^ mice, intestinal infection with Gram‐negative bacteria triggers inflammatory signaling and impaired mitophagy, resulting in abnormal exposure of mitochondrial antigens to the immune system, CD8^+^ T‐cell activation, and immune‐mediated DA neurodegeneration accompanied by motor deficits [60]. Similarly, enrichment of sulfate‐reducing bacteria, such as *Desulfovibrio*, in PD is associated with increased intestinal production of hydrogen sulfide, oxidative stress, and α‐synuclein aggregation in the gut. Experimental exposure to these bacteria or their metabolites induces enteric α‐synuclein pathology, systemic inflammatory responses, and—via gut–brain signaling—subsequent α‐synuclein accumulation in brain neurons, DA neurodegeneration, and motor deficits in animal models [61]. Importantly, gut microbiota from PD patients can transfer disease‐relevant phenotypes when transplanted into susceptible hosts. These effects originate in the intestine but lead to systemic immune activation and secondary neuroinflammatory and motor abnormalities in the brain, demonstrating that microbial communities themselves can modulate Parkinsonian disease expression [54].

Earlier infectious hypotheses of PD provide historical context for current microbiome‐based models. In particular, *Nocardia asteroides*, a soil‐dwelling bacterium, was proposed as a potential trigger of Parkinsonian pathology based on animal studies showing delayed, selective DA neuron loss and motor abnormalities following systemic exposure, even in the absence of persistent infection [62]. Initial serological studies suggested elevated immune responses to *Nocardia* antigens in small Parkinson’s cohorts, raising the possibility of prior exposure [63]. However, subsequent larger case–control studies failed to confirm a consistent association, and postmortem analyses did not identify *Nocardia* organisms in Parkinsonian brains, with later work indicating potential antibody cross‐reactivity with related actinomycetes rather than true infection [64, 65]. Although not supported as a direct cause, these studies introduced the concept that transient peripheral infection may initiate delayed, immune‐mediated neurodegeneration—an idea now echoed in contemporary gut microbiome and immune‐priming models of PD. Epidemiological evidence further suggests that immune training through vaccination is associated with reduced dementia risk, supporting the concept that modulation of systemic immune tone can influence long‐term neurodegenerative outcomes [66]. Similar protective associations have been reported for herpes zoster, pegivirus, pneumococcal, and tetanus–diphtheria–acellular pertussis (Tdap) vaccinations in older adults, reinforcing the link between peripheral immune modulation and neurodegenerative risk [67].

Viral exposures may contribute through analogous mechanisms. Although no virus has been shown to directly cause PD, multiple lines of evidence suggest that viral infections can establish long‐lasting neuroimmune alterations. Historical associations between influenza and postencephalitic parkinsonism, together with experimental data showing influenza‐induced microglial activation and α‐synuclein aggregation in animal models, support a role for viral‐triggered neuroinflammation [68]. More recently, human pegivirus has been detected in a substantial fraction of PD brains but not in controls, where its presence correlates with altered immune signaling and disease severity, suggesting that persistent or latent viral infection may exacerbate neurodegenerative processes without acting as a primary neurotropic pathogen [69].

Environmental exposures further converge on these immune pathways. Pesticides, industrial pollutants, heavy metals, and air pollution have all been associated with increased PD risk, particularly in geographically clustered populations. These factors induce oxidative stress, mitochondrial dysfunction, and chronic immune activation—mechanisms that overlap with microbial and viral immune priming and may lower the threshold for α‐synuclein misfolding and neuronal injury [51, 70]. From this perspective, PD emerges as the consequence of long‐term failure to resolve immune and metabolic stress rather than the inevitable outcome of inherited mutations.

Taken together, current evidence supports a model in which PD arises predominantly from environmental and immunological pressures acting on a susceptible biological background. Microbial dysbiosis, infectious exposures, and environmental toxins may act as initiating or amplifying factors that drive chronic inflammation, mitochondrial stress, and progressive neurodegeneration. This framework aligns with clinical observations of long prodromal phases, multisystem involvement, and substantial variability in disease onset and progression, and it highlights immune–environment interactions as critical control points for prevention and disease modification.

## 4. Immune Dysregulation as a Convergent Feature of PD

PD is increasingly recognized as a disorder of chronic, dysregulated immune activation rather than isolated neuronal degeneration. Although classical descriptions emphasize DA neuron loss and basal ganglia circuit dysfunction, large clinical and experimental studies demonstrate early and persistent engagement of both innate and adaptive immune pathways throughout disease progression [1, 2, 5]. Converging evidence indicates that PD is characterized by early and sustained neuroimmune activation, including microglial activation, elevated proinflammatory cytokines such as IL‐1β, TNF‐α, and IFN‐γ, alterations in peripheral immune cell populations, and vascular immune signaling, with many of these changes detectable in brain tissue and biofluids prior to the onset of overt motor symptoms [5, 35, 71, 72].

Multiple, partially independent factors contribute to this immune dysregulation. Misfolded α‐synuclein acts as a potent innate immune stimulus, activating microglia and inflammasome pathways through pattern‐recognition receptors such as TLR2 and TLR4, thereby sustaining local cytokine production and synaptic dysfunction [5, 73]. In parallel, mitochondrial dysfunction and oxidative stress—hallmarks of vulnerable DA neurons—promote metabolic failure, reactive oxygen species generation, and release of damage‐associated molecular patterns (DAMPs), further amplifying immune activation [5, 71]. Proinflammatory cytokines released during chronic neuroinflammation, including IL‐1β, TNF‐α, and IFN‐γ, disrupt synaptic transmission by altering excitatory–inhibitory balance, impairing synaptic plasticity, and interfering with glial metabolic support, thereby linking immune activation to circuit dysfunction and neuronal vulnerability in PD [5, 71].

Peripheral immune influences also play a significant role. Systemic inflammation communicates with the brain through multiple complementary mechanisms, including cytokine transport and signaling across the blood–brain barrier (BBB), direct sensing by circumventricular organs and the choroid plexus, neural transmission via afferent vagal pathways, and inflammation‐induced endothelial activation. Circulating cytokines can activate brain endothelial cells and glia without overt barrier breakdown, while chronic inflammatory states promote vascular dysfunction, altered neurovascular coupling, and limited immune cell trafficking. Together, these pathways convert peripheral immune activation into sustained neuroinflammatory and metabolic stress responses that impair synaptic function and increase neuronal vulnerability [5, 71]. Gut dysbiosis, increased intestinal permeability, and altered microbial metabolites can drive systemic inflammation and prime neuroimmune responses, as demonstrated in both human studies and animal models [54, 55, 60]. Aging adds an additional layer of vulnerability through immunosenescence, characterized by chronic low‐grade inflammation and progressive weakening of immune regulatory restraint. Experimental studies demonstrate that central nervous system immune checkpoints normally limit microglial inflammatory activation, while aging is associated with reduced neuronal capacity to restrain cytotoxic immune responses, thereby amplifying inflammatory stress in PD [5, 71, 74, 75]. Importantly, these inflammatory cascades are not fixed but dynamically regulated and potentially reversible. Experimental studies demonstrate that modulation of microglial activation states, inhibition of inflammasome signaling, and restoration of metabolic balance can attenuate neuroinflammatory damage and improve neuronal survival [76, 77]. These findings support the concept that neuroimmune dysregulation represents a modifiable component of PD rather than merely an irreversible consequence of neuronal degeneration. Together, these processes establish a permissive inflammatory environment in which neuronal resilience is progressively eroded.

Within this multifactorial immune landscape, an additional factor—Aβ production and deposition—should be viewed as one of several context‐dependent immune modulators. Factors such as α‐synuclein pathology, metabolic stress, vascular dysfunction, peripheral immune activation, and aging collectively shape neuroimmune tone and disease trajectory. Against this background, Aβ emerges as an injury‐ and inflammation‐responsive peptide that can further modulate immune signaling under specific conditions, particularly those involving platelet activation and vascular stress.

## 5. Aβ in Parkinsonism and the Platelet Immune Hypothesis

Aβ pathology is increasingly recognized in PD, particularly in patients who develop mild cognitive impairment or dementia. However, in contrast to Alzheimer’s disease, where Aβ accumulation is extensive and central to disease definition, Aβ involvement in PD is heterogeneous, regionally restricted, and inconsistently associated with motor severity or global cognitive decline. Major clinical reviews of PD emphasize DA degeneration, α‐synuclein pathology, and network dysfunction, while acknowledging—but not fully integrating—immune activation, vascular stress, and peripheral contributors [1, 2]. This gap has contributed to the persistent view of Aβ as a secondary or incidental finding in PD rather than a biologically meaningful immune signal.

A critical distinction relevant to Parkinsonism is the molecular form of Aβ present. Under physiological and inflammatory conditions, Aβ is predominantly presented as small soluble assemblies—most commonly dimers and low‐order oligomers—while true monomeric species are transient and rare [78, 79]. With increasing local concentration, impaired clearance, or sustained inflammatory signaling, these soluble assemblies undergo stepwise self‐association into protofibrils and fibrils, which may subsequently accumulate into plaque‐like deposits [80]. Importantly, soluble oligomers, fibrillar aggregates, and plaques are related to distinct cellular responses: soluble species readily interact with immune receptors and synapses, whereas fibrils and plaques are preferentially recognized and processed by microglia, astrocytes, endothelial cells, and perivascular macrophages through phagocytic and containment mechanisms [81].

This distinction in Aβ assembly has direct implications for PD imaging and the interpretation of Aβ burden. Amyloid PET tracers bind primarily to fibrillar and plaque‐associated Aβ, whereas soluble oligomeric species are largely invisible to current imaging modalities. Consequently, a negative or low‐signal amyloid PET scan does not exclude biologically relevant Aβ activity in PD, particularly when Aβ is present predominantly in soluble or rapidly cleared forms. Imaging studies in PD consistently show lower overall amyloid signal than in AD, supporting the concept that the equilibrium in PD is shifted toward soluble and transient Aβ species, while in AD it is shifted toward persistent fibrillar accumulation and plaque deposition [82–85]. These quantitative and kinetic differences likely explain why Aβ contributes variably to cognitive outcomes in PD while remaining central in AD.

An often underappreciated contributor to Aβ biology in Parkinsonism is its peripheral and vascular origins. Platelets are the dominant systemic source of amyloid precursor protein (APP) and circulating Aβ, accounting for approximately 90% of plasma Aβ under physiological conditions [86]. Platelets store APP in α‐granules and rapidly release it upon activation during thrombosis, inflammation, or vascular injury. Crucially, endothelial cells express secretases that cleave platelet‐derived APP, generating Aβ at the vascular interface and in surrounding tissues [87]. In addition, platelets function as immune effector cells, expressing pattern‐recognition receptors, releasing cytokines and antimicrobial peptides, and actively shaping innate immune responses [88–90].

Experimental data support the concept that platelet‐derived Aβ is generated acutely and locally in response to tissue injury. Platelet activation during thrombosis leads to rapid Aβ release and local accumulation in peripheral tissues such as skin [91]. More recently, focal DA injury and microtrauma in the rat substantia nigra were shown to produce localized increases in brain Aβ concentrations, consistent with injury‐associated generation rather than slow, plaque‐driven accumulation [92]. These findings indicate that Aβ can be produced in the brain as part of an immediate inflammatory and vascular response to neuronal damage, without requiring classical amyloidogenic cascades typical of AD.

From an immunological perspective, Aβ functions as an innate immune peptide. Soluble Aβ exhibits antimicrobial activity against bacteria, fungi, and viruses and can aggregate around pathogens, disrupt microbial membranes, and limit infection [93, 94]. In PD, where chronic neuroinflammation, endothelial dysfunction, platelet activation, and BBB stress are common, repeated release of platelet‐derived Aβ may represent a physiological immune response that becomes maladaptive when sustained. Soluble Aβ can activate microglia and amplify cytokine production, reinforcing inflammatory loops in regions already vulnerable due to α‐synuclein pathology and metabolic stress [5, 95]. At the neurovascular interface, Aβ dynamics are tightly regulated by interactions among endothelial cells, platelets, and immune receptors. Endothelial dysfunction can impair Aβ clearance and promote local inflammatory signaling, while platelet activation provides a rapid source of APP and Aβ during vascular injury [86]. Receptor‐mediated pathways involving pattern‐recognition receptors and Fc receptors further help determine whether Aβ signaling remains protective or transitions into a proinflammatory state [95]. These mechanisms highlight the importance of vascular–immune coupling in shaping Aβ‐related responses in PD.

Taken together, Aβ in Parkinsonism is best conceptualized not as a primary toxic driver, but as a context‐dependent immune effector and marker of inflammatory and vascular stress. Differences between PD and AD likely reflect shifts in the balance between soluble and insoluble Aβ species rather than fundamentally distinct biological roles. This framework reconciles the weak correlation between amyloid imaging and clinical severity in PD with strong evidence for immune activation, platelet involvement, and vascular dysfunction, and aligns PD with a broader class of neurodegenerative disorders in which innate immune mechanisms transition from protective to pathological when chronically engaged. This interpretation is consistent with imaging and neuropathological studies showing that amyloid burden in PD is highly heterogeneous, often modest, and most strongly associated with cognitive impairment when it reflects concomitant Alzheimer‐type pathology rather than primary Parkinsonian neurodegeneration [85, 96, 97].

These observations position Aβ as a persistent innate immune signal that can intersect with immune checkpoint regulation, including programmed death receptor 1/programmed death ligand 1 (PD‐1/PD‐L1) pathways that modulate inflammatory responses, and with NAD^+^‐dependent metabolic resilience in vulnerable neuronal populations.

## 6. Immunometabolic Integration of Aβ, Immune Checkpoints, and NAD^+^ Homeostasis

PD‐1/PD‐L1 signaling represents a critical immune homeostatic checkpoint that limits excessive inflammation in the central and peripheral nervous systems, regulating effector T‐cell and microglia/macrophages responses and maintaining immune tolerance under conditions of chronic immune activation [73, 98]. In PD, several established pathogenic factors, such as α‐synuclein aggregation, chronic microglial activation, mitochondrial dysfunction, gut‐derived immune priming, vascular stress, and aging, converge to generate sustained proinflammatory cytokine environments dominated by IFN‐γ, TNF‐α, and IL‐1β [5, 71]. These cytokines are potent regulators of PD‐1 and PD‐L1 expression through signal transducer and activator of transcription 1 (STAT1)–interferon regulatory factor 1 (IRF1) and nuclear factor kappa beta (NF‐κB)‐dependent pathways, making immune checkpoint regulation an important node integrating inflammatory burden with cellular stress responses [5, 99, 100].

Within this broader context, a unifying immunometabolic link between amyloid biology and immune checkpoint signaling emerges when Aβ is viewed as an injury‐ and inflammation‐responsive innate immune peptide. Platelets are the major systemic reservoir of APP/Aβ and release APP rapidly during inflammatory activation, with local conversion to Aβ occurring in peripheral tissues and at vascular interfaces [86, 87, 91]. In parallel, platelets function as immune effector cells that express pattern‐recognition receptors and amplify inflammatory cascades [88–90]. Because PD features chronic microglial activation and vascular stress, platelet activation provides a plausible mechanism for repeated, localized bursts of Aβ generation during tissue damage and inflammation, consistent with our observations that nigral injury/microtrauma in the rat model increases local Aβ concentrations in the affected brain region [92] and that thrombosis drives Aβ release in peripheral tissues [91]. Soluble Aβ oligomers activate innate immune receptors, such as TLR2, on microglia and peripheral immune cells, triggering NF‐κB signaling and the release of proinflammatory cytokines, including TNF‐α [95]. Inflammatory cytokines, particularly IFN‐γ, are potent inducers of PD‐L1 expression via STAT1–IRF1 and NF‐κB–dependent transcriptional programs that require intact NAD^+^/NAMPT metabolism [99, 100]. Checkpoint pathways are relevant to PD development, as PD‐1/PD‐L1 signaling is a core regulator of inflammatory amplitude, which can influence brain tissue injury responses and neuroinflammation; microglia often act as suppressors by expressing PD‐L1 [101, 102]. Additionally, it was shown that neuronal‐enriched extracellular vesicles trigger PD‐L1‐mediated T‐cell suppression in PD [103].

Importantly, PD‐1/PD‐L1 activity is not static across the lifespan. A recent study reports that neuronal PD‐L1 expression declines with age in humans and mice, correlating with increased CD8^+^ T‐cell accumulation in the aging brain. In that work, restoring neuronal PD‐L1 in aged mice via adeno‐associated virus transduction reduced neuroinflammation and rescued cognitive decline (Jin et al. [75]). This report is currently a preprint. This aging‐related checkpoint decline provides a mechanistic rationale for why immune checkpoint perturbation, either by aging or by therapeutic blockade, might destabilize neuroimmune homeostasis and thereby modulate neurodegeneration‐associated pathways, including Aβ biology.

It was shown that a key mechanistic bridge between immune checkpoints and inflammatory metabolism may be mediated by NAD^+^ homeostasis, regulated by the NAMPT salvage pathway. NAMPT‐dependent NAD^+^ availability constrains inflammatory transcriptional programs (e.g., via SIRT1/2 and NF‐κB acetylation status) and shapes PD‐L1 inducibility, while inflammatory cytokines released during PD‐L1 blockade can drive glycolytic reprogramming that consumes NAD^+^ and induces compensatory NAMPT expression [99, 100, 104–106]. In neurodegenerative settings, where oxidative stress, DNA damage, and cytokine exposure can deplete NAD^+^, this PD‐1/PD‐L1 ↔ NAMPT/NAD^+^ coupling may determine whether inflammatory responses remain controlled (repair‐promoting) or become self‐amplifying (injury‐promoting). Emerging evidence indicates that immune checkpoint signaling is tightly integrated with cellular metabolism. PD‐1/PD‐L1 pathways are influenced by metabolic states and NAD^+^ availability, while metabolic stress can alter checkpoint expression and function [99, 107]. In parallel, NAD^+^‐dependent enzymes such as sirtuins regulate inflammatory transcriptional programs, including NF‐κB signaling, thereby linking cellular energy status to immune regulation [108, 109]. This bidirectional relationship suggests that interventions targeting NAD^+^ homeostasis may indirectly modulate immune checkpoint activity and influence neuroinflammatory outcomes in neurodegenerative disease. Thus, Aβ release during inflammation, checkpoint regulation of immune effector intensity, and NAD^+^‐dependent immunometabolic buffering can be conceptualized as a single axis governing whether inflammation resolves or evolves into chronic tissue damage.

## 7. Clinical Neurological Manifestations of Immune Checkpoint Modulation

Clinically, disruption of PD‐1/PD‐L1 signaling via immune checkpoint inhibitors highlights both the power and the risks of checkpoint manipulation in the nervous system. Neurological immune‐related adverse events (n‐irAEs) are uncommon (about 7% of cases) but well documented and include encephalitis, movement disorders, and cognitive symptoms [110–112]. In a clinical setting, anti‐PD‐L1 monoclonal antibodies (mAbs), such as pembrolizumab (Keytruda) and nivolumab (Opdivo), are used, while new small‐molecule PD‐L1 inhibitors are currently in development. Antibodies (large molecules) have difficulty penetrating the intact BBB, whereas small‐molecule PD‐L1 inhibitors show promise for improved BBB permeability and treatment efficacy, with greater potential to exacerbate neuroinflammation and related neurodegenerative disorders. With anti‐PDL1 mAb, prospective patient‐reported outcome studies demonstrate that clinically significant cognitive impairment can occur during therapy, often peaking within the first months after initiation; however, reports of a progressive “dementia syndrome” as a dominant outcome remain comparatively rare relative to transient or subacute cognitive symptoms [113]. In contrast, there are clear case‐based and systematic signals that immune checkpoint inhibitors can precipitate Parkinsonism, including immune checkpoint inhibitor–triggered Parkinsonism in metastatic melanoma and immune checkpoint inhibitor–associated striatal encephalitis presenting with progressive Parkinsonism, as well as broader autoimmune movement disorder phenotypes under checkpoint blockade [114–117]. Together, these clinical observations reinforce the central premise of this review: PD‐relevant neurodegenerative phenotypes can be influenced by the PD‐1/PD‐L1–NAMPT–NAD^+^ axis, a particularly high‐leverage node that couples immune restraint to energy metabolism.

These considerations position NAMPT‐dependent NAD^+^ salvage as a central immunometabolic control point at the intersection of innate immune signaling, immune checkpoint regulation, and neuronal survival. In PD, chronic inflammatory activation, vascular stress, and immune checkpoint dysregulation converge to deplete NAD^+^ and compromise mitochondrial function, redox balance, and DNA repair capacity in vulnerable neurons [5, 73, 118, 119]. Pharmacological strategies that selectively restore stress‐depleted NAD^+^, therefore, represent a rational approach to stabilizing neuroimmune homeostasis. In this context, small‐molecule NAMPT modulators such as P7C3‐A20 provide a mechanistically grounded example of how targeting a single immunometabolic node may simultaneously buffer inflammatory signaling, preserve neuronal energy balance, and increase resistance to programmed cell death across multiple neurodegenerative settings.

## 8. Targeting NAD^+^ Salvage to Stabilize Neuroimmune Homeostasis

The convergence of innate immune activation, immune checkpoint regulation, and neuronal vulnerability on NAD^+^ availability identifies NAMPT‐dependent NAD^+^ salvage as an important immunometabolic control point in PD. Chronic inflammation, DNA damage, and pathological cytokine signaling, common to PD, are all potent drivers of NAD^+^ depletion [5, 73, 118, 119]. NAD^+^ is an essential redox cofactor supporting glycolysis, the tricarboxylic acid cycle, and mitochondrial oxidative phosphorylation. Depletion of NAD^+^ disrupts the NAD^+^/NADH redox couple, impairs electron transfer, and predisposes cells to oxidative imbalance and stress [120]. Loss of NAD^+^ compromises mitochondrial oxidative metabolism, redox balance, epigenetic regulation, and cellular stress resilience, thereby lowering the threshold for programmed neuronal death in DA and other vulnerable neuronal populations. In humans, dietary vitamin B3 precursors (nicotinic acid, nicotinamide, and related forms) contribute only a minor fraction of total NAD^+^ requirements through de novo and Preiss–Handler pathways, accounting for approximately 1%–2% of daily demand. Instead, cellular NAD^+^ homeostasis is maintained predominantly by the nicotinamide salvage pathway, which regenerates ∼80%–90% of NAD^+^ by recycling nicotinamide (NAM) produced through continuous NAD^+^ consumption by sirtuins, poly(ADP‐ribose) polymerases, and CD38. In this pathway, NAM is converted by the rate‐limiting enzyme NAMPT to nicotinamide mononucleotide (NMN) and subsequently back to NAD^+^, allowing the intracellular NAD^+^ pool to turn over multiple times per day. Disruption of this salvage cycle by inflammation, DNA damage, or metabolic stress, therefore, leads to progressive NAD^+^ depletion, impaired bioenergetic and redox control, and heightened neuronal vulnerability [109, 118, 120, 121], presenting NAMPT as the bottleneck enzyme in this process [122]. Therapeutic strategies that restore stress‐depleted NAD^+^ without inducing supraphysiological metabolic activation are therefore well aligned with the immunometabolic framework described above, because they may simultaneously buffer inflammatory signaling, preserve mitochondrial function, and improve neuronal resistance to immune‐mediated injury.

P7C3‐A20, a member of the aminopropyl carbazole family, provides a mechanistically grounded example of such an approach. P7C3‐A20 acts as a positive allosteric modulator of NAMPT, the rate‐limiting enzyme in the NAD^+^ salvage pathway, thereby enhancing conversion of nicotinamide to NMN and replenishing intracellular NAD^+^ pools under conditions of metabolic stress [123]. Restoration of NAD^+^ via NAMPT activation enhances SIRT1/3 activity, reinforcing mitochondrial function, redox balance, and neuronal resistance to metabolic and inflammatory stress, thereby promoting survival of vulnerable DA neurons [123, 124]. Importantly, P7C3‐A20 does not drive NAD^+^ above physiological ceilings but instead preferentially restores NAD^+^ levels depleted by injury, inflammation, or excessive DNA damage responses [124]. This property distinguishes it from simple NAD^+^ precursor supplementation and positions it as a homeostatic stabilizer rather than a metabolic stimulant.

Across multiple experimental systems, P7C3‐A20 has demonstrated robust neuroprotective efficacy. In toxin‐based models of PD, including 6‐hydroxydopamine–induced DA injury, P7C3 compounds preserve substantia nigra neurons and attenuate motor deficits, consistent with enhanced neuronal resistance to stress‐induced death pathways [124]. In models of traumatic brain injury and ischemic stroke, P7C3‐A20 reduces axonal degeneration, limits BBB disruption, suppresses microglial activation, and improves cognitive outcomes even when administered after injury onset. Primate studies further confirm neuroprotective effects within hippocampal circuitry, supporting translational relevance [125].

Recent work extends these findings to advanced neurodegenerative pathology, demonstrating that pharmacologic enhancement of NAMPT activity can reverse multiple convergent disease features, including mitochondrial dysfunction, oxidative stress, neuroinflammation, vascular impairment, and cognitive decline, in mouse models with established Alzheimer‐like pathology [126]. These effects are strictly NAMPT‐dependent, as blockade with the NAMPT inhibitor FK866 abolishes protection, confirming NAMPT as the critical mediator. At the cellular level, restored NAD^+^ availability engages mitochondrial sirtuin pathways (notably SIRT3), stabilizes energy metabolism, and constrains inflammatory gene expression, thereby buffering immune‐driven metabolic stress [123, 126].

Several other small molecules enhance NAD^+^ biosynthesis by positively modulating NAMPT, the rate‐limiting enzyme of the salvage pathway. The aminopyridine analog P7C3‐S243, a polar derivative of P7C3‐A20, exhibits superior neuroprotective efficacy compared with P7C3‐A20 in experimental models of PD, amyotrophic lateral sclerosis (ALS), and traumatic brain injury [127]. The small molecule SBI‐797812 potently activates NAMPT, shifting the enzymatic equilibrium toward NMN and NAD^+^ production; it increases catalytic activity by more than twofold in vitro and elevates intracellular NMN (∼2.5‐fold) and NAD^+^ (∼1.25‐fold) [128]. Additional NAMPT positive allosteric modulators (PAMs), including NP‐A1 and NP‐A3 series compounds, enhance ATP affinity, stabilize the phosphorylated His247 intermediate, and promote NMN formation, with reported neuroprotective and anti‐oxidative effects [129].

Among plant‐derived compounds, Myricitrin directly enhances NAMPT catalytic activity at micromolar concentrations, approximately doubling enzymatic output [130]. By contrast, dihydromyricetin (DHM, ampelopsin) has not been directly demonstrated to act as a NAMPT allosteric activator. However, DHM consistently activates AMPK and SIRT1/3 signaling pathways, reduces oxidative stress, and improves mitochondrial function in neurodegenerative contexts [131, 132]. In PD‐relevant models, including a Dicer conditional knockout mouse model, DHM alleviates motor dysfunction and prevents DA neuron loss [133].

Although direct NAMPT activation by DHM has not been established, its structural similarity to myricitrin and its modulation of NAD^+^‐dependent pathways raise the possibility that partial effects on NAD^+^ homeostasis may contribute to its neuroprotective actions. Whether DHM influences NAMPT activity or intracellular NAD^+^ levels directly remains to be experimentally verified.

By reinforcing NAD^+^ homeostasis downstream of immune activation, platelet‐derived inflammatory signaling, and immune checkpoint perturbation, NAMPT modulation offers a means to decouple necessary immune responses from progressive metabolic collapse. In the absence of a real cause of PD in particular individuals, rather than targeting α‐synuclein aggregation, dopamine deficiency, or inflammation in isolation, this strategy addresses a shared vulnerability: the failure of energetic and redox buffering under chronic immune stress. As such, P7C3‐A20 exemplifies how targeting immunometabolic control points may yield disease‐modifying benefits across heterogeneous Parkinsonian phenotypes.

Schematic overview illustrating major, interconnected immunological processes implicated in PD pathogenesis. Environmental stressors, α‐synuclein pathology, metabolic stress, vascular dysfunction, peripheral immune activation, and aging, plus local generation of Aβ, function as an innate immune signal that enhances cytokine production and engages PD‐1/PD‐L1 immune checkpoint signaling. This pathway is metabolically constrained by the availability of NAD^+^ via NAMPT‐dependent salvage mechanisms. Impaired regulation of this immunometabolic axis favors persistent neuroinflammation and contributes to neuronal vulnerability and degeneration.

## 9. Conclusion: Immunometabolic Control Points as a Therapeutic Paradigm in PD

**FIGURE 1:**
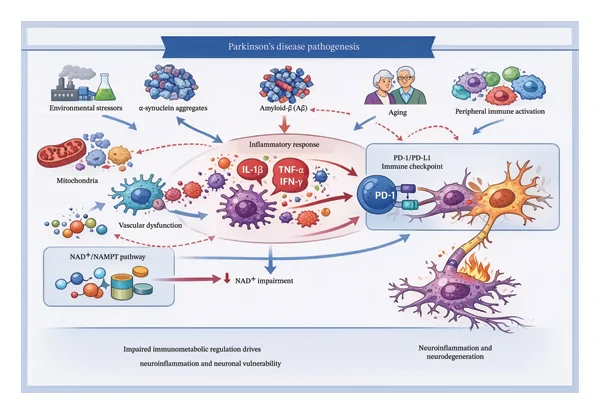
Schematic overview illustrating major, interconnected immunological processes implicated in Parkinson’s disease pathogenesis. Environmental stressors, α‐synuclein pathology, metabolic stress, vascular dysfunction, peripheral immune activation, and aging, plus local generation of amyloid‐β (Aβ), function as an innate immune signal that enhances cytokine production and engages PD‐1/PD‐L1 immune checkpoint signaling. This pathway is metabolically constrained by the availability of nicotinamide adenine dinucleotide (NAD^+^) via NAMPT‐dependent salvage mechanisms. Impaired regulation of this immunometabolic axis favors persistent neuroinflammation and contributes to neuronal vulnerability and degeneration.

PD is increasingly recognized as a disorder of failed immunometabolic regulation rather than a condition driven solely by DA neuron loss. Evidence reviewed here supports a model in which chronic, unresolved immune activation, arising from environmental exposures, microbial and viral immune priming, vascular stress, and aging‐associated immune checkpoint decline, interacts with metabolic vulnerability to drive progressive neurodegeneration (Figure 1). Within this framework, motor circuit dysfunction, prodromal nonmotor symptoms, amyloid co‐pathology, platelet activation, and immune checkpoint signaling represent interconnected manifestations of a shared pathological process rather than independent disease features.

Mechanistically, inflammation‐induced release of Aβ, largely derived from neuronal or platelet APP, can engage immune checkpoint pathways that couple immune regulation to cellular energy metabolism. Soluble Aβ oligomers activate innate immune receptors, such as TLR2 and FcγRIIb, on microglia and peripheral immune cells, promoting the production of proinflammatory cytokines, including IFN‐γ and TNF‐α. These cytokines are potent inducers of PD‐L1 expression via STAT1–IRF1 signaling and NF‐κB–dependent transcriptional programs [95, 99]. PD‐1/PD‐L1 signaling, in turn, is tightly linked to NAD^+^ metabolism: NAMPT‐dependent NAD^+^ salvage sustains PD‐L1 expression and immune tolerance, whereas NAD^+^ depletion, through inflammatory PARP activation or metabolic stress, enhances NF‐κB acetylation and further upregulates PD‐L1 as a compensatory immune‐restraining mechanism [100, 106]. With aging, neuronal PD‐L1 expression declines, weakening local immune checkpoint control and permitting CD8^+^ T‐cell–mediated neuroinflammation, a process shown to exacerbate neurodegeneration and cognitive decline (Jin et al. [75] preprint). In this framework, Aβ acts as an upstream innate immune signal that drives cytokine release, immune checkpoint engagement, and NAD^+^‐dependent metabolic reprogramming; failure of this Aβ–PD‐1/PD‐L1–NAMPT axis to resolve inflammation may shift a protective immune response toward chronic neuroinflammatory damage in PD.

This perspective highlights immunometabolic control points as particularly high‐leverage targets for disease modification. Control points such as platelet‐mediated innate immune responses, PD‐1/PD‐L1 checkpoint signaling, and NAD^+^ salvage via NAMPT regulate the amplitude, duration, and resolution of inflammatory responses while simultaneously constraining cellular energy balance, redox state, and stress resistance. Dysregulation at these nodes can shift protective immune responses toward chronic, self‐amplifying injury, rendering DA and other vulnerable neuronal populations susceptible to degeneration. Recent pharmacological and experimental evidence further suggests that such control points are modifiable: interventions (including even nutritional interventions) that dampen maladaptive inflammation, improve mitochondrial efficiency, or restore NAD^+^ homeostasis can reduce tissue injury and enhance neuronal resilience [5, 20, 134].

Therapeutic strategies that stabilize these control points, rather than suppressing single downstream pathways, offer a unifying approach to PD intervention. Modulating immune checkpoints without provoking excessive inflammation, restoring stress‐depleted NAD^+^ without inducing supraphysiological metabolic states, and limiting maladaptive platelet‐driven inflammatory signaling may collectively preserve neuroimmune homeostasis. Framing PD through this lens provides a coherent explanation for its multisystem nature, long prodromal phase, and clinical heterogeneity and suggests that effective disease‐modifying therapies will likely emerge from targeting immune–metabolic integration rather than isolated molecular lesions.

**Nomenclature**
Aβ: Amyloid‐β
AD: Alzheimer’s disease
APP: Amyloid precursor protein
BBB: Blood–brain barrier
COMT: Catechol‐O‐methyltransferase
DA: Dopaminergic
DBS: Deep brain stimulation
FcγRIIb: Fc gamma receptor IIb
GABA: γ‐Aminobutyric acid
GPe: Globus pallidus externus
GPi: Globus pallidus internus
HIF‐1α: Hypoxia‐inducible factor‐1 alpha
IL‐1β/IL‐6: Interleukin‐1 beta/interleukin‐6
MAO‐B: Monoamine oxidase B
NAMPT: Nicotinamide phosphoribosyltransferase
NAD^+^
: Nicotinamide adenine dinucleotide
NF‐κB: Nuclear factor kappa‐light‐chain‐enhancer of activated B cells
PD: Parkinson’s disease
PD‐1: Programmed cell death protein 1
PD‐L1: Programmed death‐ligand 1
REM: Rapid eye movement
SNc: Substantia nigra pars compacta
SNr: Substantia nigra pars reticulata
STN: Subthalamic nucleus
TLR2: Toll‐like receptor 2
TNF‐α: Tumor necrosis factor alpha
VOC: Volatile organic compound

## Funding

This work was supported by the National Institute of General Medical Sciences (NIGMS), National Institutes of Health (NIH) under grants SC3GM143983 , R16GM153522 and P50GM133807, and by the National Cancer Institute (NCI), National Institutes of Health (NIH) under grant R15CA287203.

## Disclosure

The content is solely the responsibility of the authors and does not necessarily represent the official views of the National Institutes of Health.

## Conflicts of Interest

The authors declare no conflicts of interest.

## Data Availability

Data sharing is not applicable to this article as no datasets were generated or analyzed during the current study.
